# Tübinger Training for Autism Spectrum Disorders (TüTASS): a structured group intervention on self-perception and social skills of children with autism spectrum disorder (ASD)

**DOI:** 10.1007/s00406-022-01537-y

**Published:** 2023-01-11

**Authors:** Laura Luisa Drüsedau, Antonia Götz, Lena Kleine Büning, Annette Conzelmann, Tobias J. Renner, Gottfried M. Barth

**Affiliations:** 1 Department of Child and Adolescent Psychiatry, Psychosomatics and Psychotherapy, University Hospital of Psychiatry and Psychotherapy, Tübingen, Germany; 2 Department of Psychology (Clinical 15 Psychology II), PFH-Private University of Applied Sciences, Göttingen, Germany

**Keywords:** Autism, ASD, Group psychotherapy, Group training, Body perception, Mindfulness, Children

## Abstract

In autism spectrum disorders (ASD), social communication and stereotypical behaviour patterns affect all areas of life, and can result in a decrease of its quality. Previous research has shown promising results for the social skills of groups of children with ASD. Furthermore, a pilot study of the Tübingen Group Training for Autism Spectrum Disorders (TüTASS) has demonstrated that mindfulness-based elements achieve additional positive effects. To build on these findings, the TüTASS training was adapted and expanded. Indeed, the TüTASS currently includes 20 90-min appointments starting with the basic skills of emotions, body, and communication, which are then transferred to personal, family, peer, and school spheres. The appointments have a fixed, consistent structure and each includes a body awareness exercise. In this study, we evaluated the TÜTASS with 27 children with ASD. The results showed improvements in pre-post comparison in behaviours associated with autism, as well as in externalising and internalising behaviours as assessed by parent reports, participant self-reports, and independent raters in participating children. Furthermore, the perceived parent burden in relation to their children decreased, whereas the participants’ self-rated quality of life increased. Overall, both the participants and their parents rated the TüTASS very positively in rating sheets and in free feedback. If replicated in larger controlled trials, TÜTASS training might be a useful treatment tool for groups of children with ASD.

## Introduction

The clinical relevance of autism spectrum disorder (ASD) has grown considerably in recent years, partly due to its ever-increasing prevalence in children and youth [1–3]. Persistent patterns of fundamental deficits in social interaction and understanding are typical [4–6]. Moreover, as stereotypies in behaviour and interests occur, the need for structured daily and life routines becomes greater. These special characteristics cause serious difficulties, especially in such social settings as schools, peer groups, or family.

The search for etiologic factors of ASD is wide-ranging. Solid evidence for a single etiological theory, or even a specific number of causes, is not yet available [5]. The etiological models in the literature are based on neurobiology and genetic principles, as well as demographic risk factors, early environmental influences, and neuropsychological theories [4–6]. The deficit in the Theory of Mind (ToM) is currently considered to be the most studied construct in the field of autism in neuropsychological research [7]. In particular, these findings are hugely important to the development of a therapeutic approach because other types of treatment (e.g., medication) have not yet been identified. Neuropsychological theories have sought to explain the reduced reception of emotions in facial expressions and body language, as this information often cannot be processed further, as well as lower levels of empathy in persons with ASD [8, 9]. In addition to the perception of other people’s feelings, the processing and regulation of one’s own emotions are often faulty [7, 10]. Thus, alexithymia is also much more common in those on the spectrum than in people without ASD [11]. A part of the described autistic symptoms can in all likelihood be attributed to sensory and motor characteristics [12–15]. The sensory characteristics tend to be present in approximately 80–90% of people with autism in the form of hypo- or hyper-reactivity to stimuli of any modality, including interoceptive stimuli. The abnormalities in motor development (e.g., lack of coordination, problems in pencil use)—and especially those that include top-down processes, such as cognitive control and executive functions—are often associated with autistic traits but have yet to be included in the diagnosis [4, 14–16].

The majority of current group interventions for ASD focus on socioemotional skills. Other well-established trainings (e.g. TOMTASS [17], SOSTA-FRA [18] or KOMSSI [19] focus on social interactional skills, but do not follow a mindfulness-based approach. However, regarding etiological factors, it seems beneficial to include mindfulness-based treatment elements as they have the potential to improve hyper- and hypo-reactivity. Indeed, not only have mindfulness-based interventions been successfully used in other disorder groups (chronic pain, anxiety, eating disorders, stress, etc.) for quite some time [20–27], initial studies in the field of autism have also demonstrated a positive effect of these therapeutic interventions. For example, Ridderinkhof et al. [28] found that mindfulness training can support adolescents on the spectrum in the regulation of their communication, emotions, and behaviour, and Gawronski et al. [29] include mindfulness-based exercises in every session of their training with autistic adults (GATE). There is also evidence to suggest that mindfulness facilitates the learning of empathy and can also increase overall well-being [30, 31], especially when participants form a homogeneous group in terms of their autistic expressions [32]. Based on these findings, we integrated the mindfulness exercises into TüTASS as part of a group therapy. Through the application in the group, the participants got the opportunity to apply and learn the exercises directly in the social setting, to then deepen them at home in the context of homework in an individual setting and to integrate them into everyday life. In addition to these factors, resource reasons also played a role in why the mindfulness exercises were not conducted in the individual setting.

Thus, while growing academic and societal interest in ASD has led to increased implementation of intervention projects in recent years, there is still a need to research more treatment strategies that additionally focus on body awareness in children.

On this basis, we developed the *Tübinger Training für Autismus-Spektrum-Störungen* (TüTASS) a few years ago and since 2014 evaluated it in an initial pilot study [33]. In the present study, we seek to combine insights from our previous training with further exercises on social skills in ASD with a strong influence of body perception content. We designed the TüTASS to help children and adolescents with an ASD cope better in their everyday lives. This can be achieved by improving social interaction and decreasing behavioural problems through the practice of mindful awareness of the body, environment, and feelings. These exercises are used to ameliorate the core problems of autistic children and adolescents. Through body awareness exercises, and on the basis of the multi-layered examination of feelings and mindful perception, the practitioners learn core competencies that can hugely support many areas of life. During the pilot study, we repeatedly reviewed the everyday needs of the participants and their parents, and included additional elements to the training. The revised training contains expanded and new components that are intended to provide support for the families of those involved. First, the important core skill of communication has become more comprehensive. Building on these basic skills, specific everyday problem areas of children and adolescents with ASD are addressed. In the process, the knowledge and skills already acquired in the areas of emotions, body, and communication are transferred to the central areas of life: self, family, friends, and school. The training also provides room for addressing the individual problems of the children and adolescents. We designed small exercises to help participants transfer the training content they have learned to their everyday lives. Based on the positive feedback and data, the elements of the pilot study were largely retained in the revision and expansion of the training, and supplemented with classic social skills elements in which everyday problems of the families are dealt with. In this second pilot study, we evaluated the revised and expanded training using pre–post surveys.

The findings from this study show improvements to emotional and behavioural problems, and that the training was very well received by both participants and parents alike. Therefore, we used the evaluation results to further improve the training and its effectiveness, feasibility, and acceptance by the parents and participants of the TüTASS programme. This has resulted primarily in changes to the context. More attention was paid to the everyday problems emphasised by participants and trainers during the evaluation. We assume that the further developed training reduces difficulties in daily life challenges caused by autistic symptomatology. To make possible changes visible, we again collected various data through self-reports and informant reporting. Additionally, data from the IDS-II scales, which were used to test participants by independent raters before and after the training, helped increase the objectivity of the results.

We expected an improvement to the core autistic symptoms, as well as to the external and internal behaviours, which was evaluated through external assessments by the parents and the self-reports of the participants. Moreover, we expected to see improvements in the objective assessment using IDS-II.

## Methods

### Participants

The participants were recruited via in-house recommendations. Additionally, many participants contacted us by e-mail because they had been recommended our training from various external sources. To participate in the study, they had to meet the following inclusion criteria: Aged between 7 and 14 years and have a primary diagnosis of ASD. Moreover, they had to have an IQ above 70 and the ability to participate in at least 70% of the training sessions, corresponding to 14 sessions. Measurements using the CBCL prior to the start of training suggest that 81% of participants showed internalizing (depressed, anxious, somatic and social problems, attention problems) and externalizing (aggressiveness and rule-breaking behavior) tendencies. When asked about medical conditions, 48.15% of participants indicated that they currently had medical conditions or developmental disabilities. A total of 30 patients participated in 5 groups (each comprised of 6 children and 2 therapists). One patient participated in less than 14 sessions due to family difficulties, one patient had an IQ below 70, and one patient showed other priority comorbidities. These patients were able to participate in the training but were not included in the statistical analyses. In addition, participants were not allowed to participate in other therapies outside of TüTASS.

The group programme was evaluated with 27 participants (23 male, 4 female, age 11.06 years, SD = 2.24 median = 10.00, variance = 5.25, age range 7–14 years). All of the participants were pre-diagnosed with an ASD. 21 of the participants were diagnosed with the Autism Diagnostic Observation Schedule (ADOS) tool, while the remaining 6 were diagnosed by a pre-treatment specialist. For 5 of these children, the scores we obtained using the Social Responsiveness Scale (SRS) and the Marburg Rating Scale for Asperger’s Syndrome (MBAS) were above the cut-off of the high range. For one participant, the parent's score was in the moderate range on both the MBAS and the SRS, so that the cut-off score for the diagnosis was not exceeded, but the diagnosis was confirmed in the clinical expert judgement. The mean IQ of the participants was 105.76 in *n* = 21 (CFT 20-R = 7, WISC-IV = 6, HAWIK-IV = 5, CFT 1-R = 2, CPM = 1). Four children took methylphenidate during training, one child was given amphetamine sulphate, one child was given Elvanse, and another took Aripiprazol and Pimpamperon. 12 of the participants attended a primary school, 1 a lower secondary school, 4 a secondary or comprehensive school, 9 a high school, and 1 participant attended a Waldorf school.

We obtained written informed consent from all of the participants and their legal guardians; participation in the study was voluntary, and data were processed pseudonymously. The study was approved by the local ethics committee of the Eberhard Karls University and the University Hospital Tübingen, and conducted in accordance with the Declaration of Helsinki.

### Intervention

We designed the TüTASS programme as an ambulant structured group training for children and adolescents with ASD. In groups of 6 children and 2 group leaders, the programme was held every two weeks (except during school holidays) in a total of 20 sessions (each lasting 90 min). This design allowed us to therapeutically accompany the participants over a whole year. There was a space of approximately one year between each child’s pre-test (before treatment) and post-test (after treatment). The sessions were structured and conducted using standardised material (a manual of the TüTASS is in progress). The programme’s goal is to help children with ASD develop a more mindful awareness and understanding of their body and emotions, as well as to improve their social understanding and interaction. Such improvements are intended to help participants (and their families) better cope with everyday life. The TüTASS also follows a systemic approach, according to which not only are participants considered as individuals, but so too are their parents. All legal guardians were offered one parent-therapist meeting before and one after the training, as well as a parenting programme of four sessions during the training. Furthermore, the parents could contact the group leader with urgent questions. In combination with special homework assignments (one exercise per session on the lesson topic and one body perception exercise), a connection between TüTASS and the everyday life of the participants should be established.

The entire training programme consisted of 10 parts (1–3 sessions each; see below). Each session included one mindfulness exercise. The sessions all shared the same sequence of events: In the welcome ritual, the children and therapists used a cube on which different feelings were illustrated in order for them to share their current emotional state. Once done, the group then dealt with the exercises which had been prepared as homework. This was followed by the introduction of a new topic in each session (as described below). A closing ritual, which included mutual feedback and a game, concluded the session. To file the worksheets and homework, participants were each given a folder. The overall TüTASS programme was as follows.

#### Part 1: introduction (session 1)

The first session gives the participants an easy and relaxed start, since getting used to new structures and people is often demanding for those with autism [34]. In addition, the processes and contents of the training are explained to the participants. A group name and a greeting ritual are discussed together. The first body awareness exercise that we practiced with the children is called ‘chocolate meditation’, in which the children are taught to eat food mindfully. This offers an easy, everyday, and enjoyable introduction to mindfulness exercises.

#### Part 2: emotions (sessions 2–4)

Difficulties in emotion regulation and resulting dysfunctional behaviour are frequently observed in patients with ASD [1, 35, 36]. Therefore, these sessions served to better perceive and recognise emotions, as well as to learn strategies for coping with emotions. To practice these skills, we worked on both theoretical and practical levels (e.g., pantomime, role-playing), facial expressions and gestures of emotions, as well as the connection between certain situations and feelings. Moreover, we addressed dealing with strong emotions—especially anger—which poses special challenges for children and young people with autism [37]. The second body awareness exercise involves a story with progressive muscle relaxation (PMR) exercises. This relaxation method helps reduce anxiety in children and adolescents, and can help with sleeping problems. Furthermore, it leads to a reduction of stress and increased concentration levels.

#### Part 3: body (sessions 5–7)

People with autism can exhibit sensory sensitivities. As mentioned earlier, it has been estimated that these occur in 80–90% of autistic people in the form of hypo- or hyper-reactivity to stimuli of any modality. Accordingly, the next part of the training concerns the ‘body’ area [4, 14–16]. First, the participants were asked to focus on their sensory perceptions. A subsequent station run was about experiencing and trying out the individual senses. To improve the recognition of their own physical comfort zone, we conducted a physical closeness-distance exercise and reflected upon it afterwards. To be more attuned to one’s own body, it is important to know exactly where certain feelings can be perceived. With the help of two yoga exercises, we discussed when and where one feels strong or weak. We then dealt with facial expressions and posture in relation to emotions in different exercises. Furthermore, we addressed what tension and relaxation are, and discussed how the former can be reduced and the latter enhances through trained physical exercises. To feel mental, in addition to physical, tension, we organised a small ‘competition’ between the groups and discussed afterwards. The accompanying body awareness exercises that we performed with the children were the yoga exercises ‘Sun Salutation with Babar’ and ‘Pizza Massage’. The first of these helps reduce stress and increase rest and relaxation, while the second is intended to provide body awareness, perception, and mutual trust with physical touch.

#### Part 4: communication (sessions 8–10)

For many people on the spectrum, only the pure content of verbal language is what constitutes communication—social or emotional signals are not perceived correctly or only with a delay [38]. Therefore, we first focused on non-verbal communication through the influence of body language with facial expressions and gestures on what is said. Different meanings of gestures were learned in a playful way and other communicative aspects (e.g., tone of voice) were included. This was followed by exercises in verbal communication: Due to their deficits in the ToM people with autism find it difficult to correctly identify the interests and goals of the other person and modulate their own speech accordingly [39]. Therefore, we emphasised tracing different parts of speech and practicing asking questions, recognising communication errors in the role play of the group leaders, and guiding the team partner confidently in an obstacle course. In terms of everyday communication, the topic of ‘small talk’ was discussed in detail with impulse questions and contacting another person was practiced on the clinic premises. Finally, we talked with the children and adolescents about the advantages and disadvantages of media communication and the use of emojis. As a further body awareness exercise, we conducted the ‘noise meditation’ exercise, which was intended to prevent automatic noise interpretation, which in turn reduced subjective overload due to constant environmental noise. We also intended this exercise to aid focus and concentration in everyday situations.

#### Part 5: myself (sessions 11–12)

These sessions were intended to encourage the participants to integrate autism into their identity in the conflict between ‘normal’ life and the diagnosis. The participants had often been confronted with stigmatisation [40], and should thus be enabled to educate others about autism to counteract the prejudices they are routinely confronted with. The introduction was about sensing one’s own limits (what is perceived as (un)pleasant?). This was followed by psychoeducation on the topic of the autism spectrum. To strengthen their self-esteem, the children and adolescents were asked to reflect on their own abilities and the strengths they perceived in themselves in different areas of life. Since many children with an ASD find it difficult to be flexible in everyday life [41, 42], we felt it important to deal with these issues. As such, we incorporated individual exercises into the lesson (i.e., changing the usual routine, changing seats). Together with the participants, we structured a daily schedule and discussed how to deal with flexibility in everyday life. The feelings experienced in the process were included. We conducted the ‘Bodyscan’ mindfulness exercise, which was designed to help people relax in stressful, painful, or worrisome situations.

#### Part 6: family (sessions 13–14)

Children and adolescents with autism pose special challenges to parents in their upbringing as well as to the entire family system [37]. These parents tend to have higher stress profiles [43–45]. Against this backdrop, we conducted the ‘family scale’ exercise in the group, which focused on both the advantages and disadvantages of family. Moreover, the issue of sibling rivalry was addressed by considering the pros and cons of belonging to different sibling positions (e.g., ‘Who gets the most attention?’). Subsequently, the topic of family was linked to the feelings that occur and boundaries were discussed. Various situations were examined and practiced in role plays. Since media use or consumption can lead to conflicts at both the parent–child [46] and sibling levels [47], we discussed the topic of media through open discussions, conveying factual knowledge, and role plays. The focus was on the children reflecting on their own media behaviour and developing a sense of appropriate media use. We used the ‘walking mediation’ mindfulness exercise here, which can help focus attention on the sensations of the body so that external stimuli are perceived as less disturbing.

#### Part 7: friends (sessions 15–16)

Autism-specific abnormalities in social skills, as well as problem behaviours, seem to hinder building and maintaining deeper, reciprocal friendships [48–52]. First, an appropriate concept of friendship should be strengthened—especially regarding its positive aspects. Based on this, the participants together worked to identify how to recognise ‘real’ and ‘false’ friends by referring back to certain situations and role plays. To strengthen the participants’ commonalities, a network of connections was created between the children (visualisation of commonalities by picking up threads). To illustrate the importance of trust in relationships, as well as to make this concept tangible for the participants, a group exercise was also conducted. Then, depending on the group, the topics of friendship, love, and sexuality were addressed in an age-appropriate manner. As a mindfulness exercise, ‘belly breathing’ was performed. This exercise can help one stay in the here and now, minimise rumination, and reduce inner restlessness and anxiety.

#### Part 8: school (sessions 17–18)

Individuals with ASD require increased support in everyday school life (e.g., with organisation and planning). They find it difficult to concentrate in the often noisy and chaotic school environment. Moreover, peer interactions (e.g., in group work or during school breaks) can pose certain challenges. Furthermore, they can have experience significant problems with changes in action sequences (which characterises everyday school life). Therefore, we also focused on the daily school routine [41, 42].

As an introduction, strategies for dealing with school problems (e.g., a lack of concentration due to high noise levels in the classroom) were developed. In addition, we focused on the topic of bullying: What is bullying? Why is bullying taking place? The participants then discussed helpful strategies for ensuring that their own boundaries are respected by others.

Children and adolescents with ASD often report finding it difficult to give presentations [53]. Accordingly, it was important to practice this in a protected setting: The participants were allowed to give a short presentation on a topic of their choice. Feedback was given and helpful strategies against nervousness were elaborated upon. Regarding the question ‘Why am I learning?’, the participants were taught that they were not learning for their teachers or parents, but for themselves and their own future, and that current effort can pay off later. The mindfulness exercise ‘The safe place’ was performed. This guided imagery exercise can contribute to a reduction of fear, as well as to an increased sense of security in unpleasant situations or with overwhelming feelings.

#### Part 9: repeat game (session 19)

We used this session to repeat and deepen all of the topics covered. We opted for a self-designed game in which the participants were divided into two groups and competed against each other in practical tasks. The participants could vote on which mindfulness exercise they wanted to repeat. To practice giving positive feedback and strengthening self-esteem, we tasked the participants with writing down positive qualities about each group member. These compliments were shared with the participants by the group leaders in the follow-up session.

#### Part 10: completion (session 20)

As a final lesson, the participants walked through a ‘barefoot park’ in teams of two. Elements of the training (including ‘mindful togetherness’, ‘perceiving and feeling one's own body’, ‘clear communication’, and ‘expression of perception’) were followed and absorbed. A practical exercise to strengthen the group (“circle of community”) followed. Finally, the participants received a certificate in recognition of their commitment to, and participation in, the training. The participants were allowed to choose the final mindfulness exercise.

### Instruments

We used standardised questionnaires to collect psychometric data from the participants and their guardians both before and after the training. These questionnaires collected data on core autistic characteristics, as well as the strengths, weaknesses, and current developmental status of the participants. Moreover, a subjective assessment of parental stress and well-being was collected from the guardians and participants. We also used objective methods to assess social-emotional competencies. For instance, we used the SRS [54] to assess changes in autistic core characteristics, and the MBAS [55] for an external assessment of core symptoms. The Child Behaviour Checklist (CBCL; [56]) and the Strength and Difficulties Questionnaire (SDQ; [57]) were used to assess the patients' competences and problems. The Inventory for the Assessment of the Quality of Life in Children and Adolescents (ILK; [58]) and the Questionnaire for Measuring the Health-Related Quality of Life in Children and Adolescents (KINDL; [59]) were used to assess the patients’ and parents’ quality of life. Furthermore, we assessed parental stress through the Parenting Stress Index (EBI; [60]), and used the Experiencing Emotions Scales (SEE; [61]) to examine patients’ emotion management and emotional intelligence. For an additional, more objective assessment, children and adolescents were assessed for social skills using the Social-Emotional Competence task domain of the Intelligence and Development Scales for Children and Adolescents (IDS-II; [62]). The testing using IDS-II was conducted by people who were not involved in the training. The SRS and IDS-II were defined as primary outcomes, while the remaining questionnaires used were secondary outcomes.

Furthermore, the feasibility and quality of the group training were evaluated by both the patients and their parents. These were recorded after the completion of the training using special self-constructed questionnaires for the TüTASS. The data collected served as feedback and a basis for the training’s further development. Since these questionnaires were collected in a data-anonymised manner, the feedback could not be assigned to individual participants (including those excluded). All feedback forms were, therefore, included in the evaluation.

### Statistical analyses

SPSS for Windows, version 28 (IBM SPSS Statistics 21; IBM Corporation) was used for statistical analysis. To check the data sets for normal distribution, we examined them with a Shapiro–Wilk test before further processing. If the data were normally distributed, we used paired t-tests to examine the change between the T0 and T1 data collection times, and Wilcoxon tests if they were not. The value of 0.05 was selected as the significance level.

Since no standardisation is as yet possible for the IDS-II, and the test evaluation for the subtests was conducted according to age groups with different scales, the relatively achieved score was calculated in percent for each child (achieved score in the subtest/maximum achievable score in this age group). This allowed us to compare the test results between the age groups.

## Results

### Psychometric assessment

Table 1 shows the results for the (sub)scales of the questionnaires before and after the completion of the training, together with the statistical data. The SRS scores for social motivation and social communication improved, while there were no significant changes to the scores for social awareness, social cognition, restricted interests, and repetitive behaviour. The CBCL variables improved in the scale of external problems, as well as in both the rule-breaking behaviour and aggressive behaviour subscales. The scale of internal problems, and the withdrawn/depressed and thought problems subscales in particular, became positive, while the social problems and attention problems subscales showed a positive trend. In contrast, somatic complaints and anxious/depressed remained the same. The SDQ showed a decrease in peer problems and a positive trend in conduct problems. The data of emotional symptoms, hyperactivity, and prosocial behaviour remained stable. The ILK showed a positive trend in the children’s data for both the quality of life and the problem score, whereas the parents’ assessments remained unaltered in these areas. The data collection using the KINDL showed no change in the various areas. The self-disclosure scales for experiencing emotions measured by the SEE were rated more positively in the experience of emotional overload, body-related symbolisation of emotion, and experience of self-control. However, there was no change to the acceptance of own emotions, experiencing lacks of emotion, imaginative symbolisation of emotions, and experience of emotion regulation. The parenting stress index (EBI) showed decreases to children’s stress, but no reductions in parental stress. The Marburg Rating Scale for Asperger’s Syndrome (MBAS) improved in the ToM and in stereotypical and inappropriate behaviour. The scale for conspicuous speech style, special interests, and motor skills showed a positive trend, whereas the scale for shared attention and joy, facial expressions, and gestures remained stable. The Intelligence and Development Scales of the IDS-II for recognising emotions showed a positive trend, and the participants improved significantly (24%) in regulating emotions and social competences (15%).

**Table 1:** Clinical measurements before (T0) and upon training completion (T1), mean ± standard deviations, statistics and effect strengths (Cohens d, Wilcoxon r)

Questionnaire	T0	T1	Statistics	Effect strength
Primary outcomes				
SRS score	81.85 ± 8.66	79.38 ± 8.22	*z* = − 1.25, *p* = 0.212, *n* = 26	*r* = 0.10
SRS SocAwa	74.38 ± 11.44	73.46 ± 7.53	*t* = 0.53, *p* = 0.600, *n* = 26	*δ* = 0.10
SRS SocCog	76.81 ± 7.77	76.23 ± 9.19	*t* = 0.46, *p* = 0.652, *n* = 26	*δ* = 0.09
SRS SocCom	85.50 ± 10.76	80.35 ± 10.94	*t* = 2.40, ***p* = 0.024**, *n* = 26	*δ* = 0.47
SRS SocMot	78.12 ± 7.79	73.04 ± 9.25	*t* = 2.99, ***p* = 0.006**, *n* = 26	*δ = *0.59
SRS ResInt	77.85 ± 9.66	76.38 ± 8.99	*z* = − 1.01, *p* = 0.315, *n* = 26	*r* = 0.02
IDS-II				
IDS-II RE	85.56 ± 16.25	92.22 ± 11.21	*z* = − 1.67, *p* = 0.092, *n* = 27	*r* = 0.03
IDS-II RG	61.60 ± 29.08	85.67 ± 18.23	*z* = − 3.95, ***p* < 0.001**, *n* = 27	*r* = 0.07
IDS-II SC	56.92 ± 24.27	71.97 ± 14.95	*z* = − 3.01, ***p* = 0.003**, *n* = 27	*r* = 0.06
Secondary outcomes				
CBCL	70.96 ± 5.44	67.58 ± 7.79	*z* = − 3.93, ***p* < 0.001**, *n* = 26	*r* = 0.08
CBCL INT	67.46 ± 6.43	64.58 ± 9.47	*t* = 2.37, ***p* = 0.026**, *n* = 26	*δ* = 0.47
CBCL EXT	65.69 ± 8.84	61.61 ± 5.79	*t* = 5.48, ***p* < 0.001**, *n* = 26	*δ* = 1.08
CBCL AD	64.62 ± 8.84	63.92 ± 9.05	*t* = 0.59, *p* = 0.555, *n* = 26	*δ* = 0.12
CBCL WD	68.12 ± 7.74	64.31 ± 9.61	*z* = − 2.94, ***p* = 0.003**, *n* = 26	*r* = 0.06
CBCL SC	59.15 ± 7.64	57.46 ± 8.31	*z* = − 1.33, *p* = 0.184, *n* = 26	*r* = 0.03
CBCL SP	67.88 ± 6.26	65.73 ± 7.21	*t* = 2.26, ***p* = **0**.033**, *n* = 26	*δ* = 0.44
CBCL TP	69.50 ± 5.87	66.92 ± 6.82	*z* = − 2.29, ***p* = 0.022**, *n* = 26	*r* = 0.04
CBCL AP	69.27 ± 8.56	67.15 ± 8.71	*t* = 1.83, *p* = 0.079, *n* = 26	*δ* = 0.36
CBCL RBB	62.65 ± 6.59	59.73 ± 5.13	*t* = 3.78, ***p* < 0.001**, *n* = 26	*δ* = 0.74
CBCL AB	66.88 ± 6.22	62.77 ± 7.54	*t* = 4.04, ***p* < 0.001**, *n* = 26	*δ* = 0.79
SDQ	19.85 ± 3.68	17.81 ± 4.94	*t* = 2.79, ***p* = 0.010**, *n* = 26	*δ* = 0.55
SDQ ESS	3.85 ± 1.89	3.58 ± 1.96	*z* = − 0.68, *p* = 0.497, *n* = 26	*r* = 0.01
SDQ CPS	4.08 ± 1.57	3.62 ± 1.58	*t* = 1.89, *p* = 0.069, *n* = 26	*δ* = 0.37
SDQ HS	6.04 ± 2.62	5.58 ± 2.82	*t* = 1.13, *p* = 0.269, *n* = 26	*δ* = 0.22
SDQ PPS	5.88 ± 1.97	5.04 ± 2.13	*t* = 2.36, ***p* = 0.026**, *n* = 26	*δ* = 0.46
SDQ PS	4.73 ± 2.05	4.69 ± 1.83	*t* = 0.12, *p* = 0.917, *n* = 26	*δ* = 0.02
ILK P QOL	18.62 ± 2.06	18.80 ± 2.99	*z* = − 0.73, *p* = 0.468, *n* = 26	*r* = 0.01
ILK P PS	2.69 ± 1.35	2.43 ± 1.58	*z* = − 1.05, *p* = 0.292, *n* = 26	*r* = 0.02
ILK C QOL	20.04 ± 3.32	21.12 ± 3.71	*z* = − 1.80, *p* = 0.072, *n* = 25	*r* = 0.04
ILK C PS	2.16 ± 1.70	1.76 ± 1.59	*z* = − 1.72, *p* = 0.085, *n* = 25	*r* = 0.03
SEE	48.23 ± 10.07	44.32 ± 13.02	*t* = 1.18, *p* = 0.251, *n* = 22	*δ* = 0.25
SEE AOE	47.86 ± 11.14	43.05 ± 9.96	*t* = 2.19, ***p* = 0.039**, *n* = 22	*δ* = 0.47
SEE EEO	50.52 ± 9.39	48.81 ± 9.91	*t* = 0.92, *p* = 0.368, *n* = 21	*δ* = 0.20
SEE ELE	44.58 ± 12.83	39.37 ± 10.46	*t* = 2.50, ***p* = 0.022**, *n* = 19	*δ* = 0.57
SEE BSE	47.48 ± 11.78	47.57 ± 11.19	*t* = − 0.04, *p* = 0.967, *n* = 21	*δ* = − 0.01
SEE ISE	44.86 ± 10.52	47.52 ± 13.24	*z* = − 0.74, *p* = 0.454, *n* = 21	*r* = 0.02
SEE EER	42.82 ± 14.95	49.32 ± 13.86	*t* = − 2.23, ***p* = 0.036**, n = 22	*δ* = − 0.48
SEE ESC	48.23 ± 10.07	44.32 ± 13.02	*t* = 1.18, *p* = 0.251, *n* = 22	*δ* = 0.25
EBI	66.81 ± 5.58	64.31 ± 9.01	*z* = − 1.74, *p* = 0.082, *n* = 26	*r* = 0.03
EBI TCS	68.50 ± 4.40	65.96 ± 7.25	*z* = − 2.32, ***p* = 0.021,** *n* = 26	*r* = 0.04
EBI TPS	61.15 ± 7.21	60.04 ± 10.55	*z* = − 0.28, *p* = 0.777, *n* = 26	*r* = 0.01
MBAS	111.28 ± 21.34	102.88 ± 21.10	*t* = 3.16, ***p* = 0.004**, *n* = 25	*δ* = 0.63
MBAS TOM	42.56 ± 8.25	39.24 ± 8.78	*t* = 3.21, ***p* = 0.004**, *n* = 25	*δ* = 0.64
MBAS ATT	25.64 ± 8.88	24.32 ± 7.79	*t* = 1.18, *p* = 0.251, *n* = 25	*δ* = 0.24
MBAS SIB	24.76 ± 5.97	22.44 ± 6.42	*z* = − 2.40, ***p* = 0.016**, *n* = 25	*r* = 0.05
MBAS SKILL	18.32 ± 5.53	16.80 ± 5.99	*t* = 1.93, *p* = 0.066, *n* = 25	*δ* = 0.39

SRS, CBCL, SDQ, ILK P, EBI scores are based on data from 26 participants, and ILK C and MBAS scores are based on 25 participants. SEE ACC, SEE EXS, and SEE EXO scores are based on 22 participants, SEE EXL, SEE ISYM, and SEE EXR scores are based on 21 participants. SEE BSYM scores are based on 19 participants. IDS-II scores are based on 27 participants

Values in bold indicate significance (α = 0.05)

*SRS* social responsiveness scale with subscales, *SRS SocAwa* social awareness, S*RS SocCog* social cognition, *SRS SocCom* social communication, *SRS SocMot* social motivation, *SRS ResInt* restricted interests and repetitive behaviour. *IDS-II* intelligence and development scales-2, *IDS-II RE* recognising emotions, *IDS-II RG* = regulating emotions, *IDS-II SC* social competence. *CBCL* child behaviour checklist, *CBCL Int* internal problems (Subscales: WD, SC, AD, SP, TP, AP), *CBCL Ext* external problems (subscales: RBB, AB), *CBCL WD* subscale withdrawn/depressed, *CBCL SC* somatic complaints, *CBCL AD* subscale anxious/depressed, *CBCL SP* social problems, *CBCL TP* thought problems, *CBCL AP* attention problems, *CBCL RBB* rule-breaking behaviour, *CBCL AB* aggressive behaviour, *SDQ* strengths and difficulties questionnaire with subscales, *SDQ ESS* emotional symptoms scale, *SDQ CPS* conduct problem scale, *SDQ HS* hyperactivity scale, *SDQ PPS* Peer problem scale, *SDQ PS* prosocial scale, *ILK* inventory for the assessment of quality of life in children and adolescents, *ILK P QOL* parents—quality of life, *ILK P PS* parents—problem score, *ILK C QOL* child—quality of life, *ILK C PS* child—problem score; *SEE* Scales for experiencing emotions, *SEE AOE* acceptance of own emotions, *SEE EEO* experience of emotional overload, *SEE ELE* experience lack of emotion, *SEE BSE* body-related symbolisation of emotion, *SEE ISE* imaginative symbolisation of emotions, *SEE EER* experience of emotion regulation, *SEE ESC* experience of self-control; *EBI* parenting stress index, *EBI TCS* total children score, *EBI TPS* total parent score; *MBAS* marburg rating scale for Asperger’s syndrome, *MBAS TOM* theory of mind, *MBAS ATT* shared attention and joy, facial expressions and gestures, *MBAS SIB* stereotypical and inappropriate behaviour, *MBAS SKILL* conspicuous speech style, special interests, motor skills

### Patient and parent evaluation

Table 2 shows that, overall, the treatment was very positively evaluated by both the children and parents.

**Table 2:** Evaluation of the specially developed questionnaires for children and parents in TüTASS after training completion, mean ± standard deviations

Questions for children	
How did you like the TüTASS?	3.78 ± 1.29
Did you enjoy going to the training?	3.41 ± 1.50
Did you have fun in the training?	3.81 ± 1.23
Were you scared or worried during the training?	4.52 ± 0.87
How do you like that there are participants in the training?	4.06 ± 0.86
How did you get along with the other participants?	4.09 ± 0.78
How did you get along with the group leaders?	4.51 ± 0.47
Do the things you learned in training help you in everyday life?	3.07 ± 1.38
How well did you understand the content of the training?	4.21 ± 0.80
Questions for parents	
How do you like the concept of the TüTASS?	4.51 ± 0.74
Were your expectations of the TüTASS met?	3.71 ± 0.91
Did your child enjoy going to the training?	3.46 ± 1.29
How do you like that the training is taking place in groups?	4.65 ± 0.83
Do you think that your child has benefited from the training?	3.57 ± 1.12
Did you sometimes pick up topics from TüTASS at home?	3.63 ± 0.87
Is your child engaging with the issues in TÜTASS?	3.40 ± 1.08
How helpful did you find the training?	3.69 ± 1.00
How helpful did you find the supplemental parent training?	3.98 ± 1.14

1 = negative, 5 = positive

The children rated the sessions focusing on body perception and everyday communication as particularly useful, whereas the parents rated the sessions on the identification of emotions, body relaxation and family as especially important. Both children and parents appreciated the session for flexibility and school in particular. Generally speaking, no session was rated negatively by the parents or participants. The participation rate of the children in the individual dates was high.

### Children reports

There was also a free writing part allowing for more individual feedback. When asked whether anything had changed or been learnt during the training, the children answered that, from their perspective, they could better communicate and deal with their classmates. They had a deeper understanding for other people, and it was easier for them to maintain friendships. They also got along better with their families and in everyday life. The participants reported that they had learned to more accurately understand and deal with their feelings, that they no longer “freak out” so quickly, that they cry less, and that they are more able to calm themselves in stressful situations by means of body awareness and mindfulness exercises. Moreover, new approaches to problems, and turning to adults more often when they arise, were mentioned, as well as the topics of love and media consumption in general. When asked what they particularly liked, the children answered that they formed friendships with other people with ASD and that they liked the creative activities in the training. Additionally, the structure and procedure of the training, as well as the content and body awareness exercises, were spoken of positively. The practical application of the content was also emphasised. The participants did not like the worksheets and associated writing in the training, and particularly disliked the disruptive behaviour of other participants. Some children said that the location of the training was too far from their homes, or that its length meant they could not pursue other hobbies during that time.

### Parental reports

Some parents reported that their children were more able to cope with their diagnosis and had learned to accept their own strengths and weaknesses. Furthermore, they indicated that their children had been taught to more effectively deal with their deficits, understand that they were not alone with these problems, and recognise their own great positive potential. They further mentioned that the children became more self-confident, more able to assess feelings in themselves and others, and could better express feelings and sensations. Parents evaluated their children as more reflective and friendlier, and that tantrums became less frequent. Parents rated the awareness exercises as being of special importance in this respect and some parents indicated both that they found the mindfulness exercises helpful for their children and that their children were able to apply them in everyday situations. Indeed, from the parents’ perspective, their children could communicate and express themselves with greater efficacy, and were more responsive to the family. For their part, the parents found it easier to understand their children, deal with their issues, and improve their teamwork skills. The parents learned that the manifestations of ASD are highly diverse. They indicated that their understanding and acceptance for the peculiarities, fears, and reactions of their children had deepened, and that they had learned to take their children more seriously and accept them as they are. Parents found a greater appreciation of their children’s positive qualities, and that they had become more relaxed and patient. They also found it very helpful to learn that other parents have similar problems and to exchange ideas with them. The parents wanted to take up the suggestions from the parent training for everyday life and deepen individual topics. They also learned of the importance of taking time to relax and unwind, as well as of being confident (and less pressured) in their methods. The parents were pleased to be included in some parts of the training. They reported that it was sometimes difficult to do additional homework on top of schoolwork. In terms of content, they missed the topics of anxiety and shopping. Furthermore, the parents would also have liked to have had more appointments for parents and siblings.

### Final feedback of children and parents

At the end of the questionnaire, the children and parents had the option to give open feedback. Many thanked us for the training and hoped for further opportunities to participate in an extended TüTASS programme.

## Discussion

The aim of the study was to evaluate the revised and expanded TüTASS training. In particular, we sought to examine its feasibility, usability, and acceptability from the perspective of participating children with ASD and their parents. This training improved symptoms and was very positively evaluated, and appreciated, by the patients and parents. A very low dropout rate of *n* = 1 in 30 participants indicates the good acceptance of this structured treatment programme. The subsequent participation of many participants in further sessions as part of TüTASS Extended reflects the subjectively perceived impact and high acceptance of the training among parents and children. Both reported positive improvements in everyday life. Only the homework was rated negatively by the participating children and parents due to the additional time required. Compared to previously established interventions in the field of ASD treatment, our study shows similar treatment effects. However, due to our novel approach to the field of ASD (i.e., the implementation of body awareness exercises), TüTASS can join the ranks of other major interventions, such as TOMTASS [17] or SOSTA-FRA [18].

Children and adolescents with autism show abnormalities—especially in social interaction, communication, and stereotypical behaviours—which often lead to difficulties in everyday situations. In terms of the diagnostic questionnaires, we found various positive tendencies. The two questionnaires, SRS and MBAS (which refer specifically to autistic traits), showed improvements in several core areas of autism. Indeed, the SRS [54] showed an improvement in social communication. Three training sessions dealt with the topic in different ways in relation to non-verbal, verbal, and everyday communication, with many practical exercises (see Part 4: Communication [sessions 8–10)]. The children found the appointment for everyday communication particularly helpful. The scale on restricted interests and repetitive behaviour showed no change. In the TüTASS, we rarely noticed the preoccupation with special interests and repetitive behaviours of participants, leading us not to treat these as problematic behaviours. In the TüTASS, special interests are validated and taught to be used as resources. In the MBAS, scales with similar foci showed improvements regarding stereotypical and inappropriate behaviour. In addition, the MBAS reported improvements in the Theory of Mind (ToM), which we attribute to the topics of the perception of emotion and friendship, as well as the exercises on bullying and the exploitative behaviour of others. The topic of family in relation to dealing with conflicts and the pros and cons of sibling positions could also strengthen abilities in the ToM. The lessons on communication concerned paying attention to the other person when choosing topics for conversation and creating balanced parts of speech. We found a positive trend for the scales regarding conspicuous speech style, special interests, and motor skills. The values for the scale for divided attention and joy, facial expressions, and gestures remained stable. However, since 7 out of 13 items of this scale referred to the age of 4–5 years, no measurable change was expected. We found a significant increase to social motivation. Again, we hypothesise that participants' positive experiences in the group—compared to many negative experiences in, for example, school—make them more eager to interact, and a higher sense of competence also increases social motivation. This effect could also be found in a group-based training in adults with autism [63]. Here in particular, as in other intervention programmes (e.g., SOSTA-FRA [18] and TOMTASS [17]), the clear advantages of a group over individual intervention are evident. The children are given the opportunity to experience the advantages of friendships and teams for themselves in a guided, well-meaning setting. While parent-reported scores for social awareness and social cognition did not change significantly, the self-report scales of the children for experiencing emotions (as measured by the SEE) showed improvements for experiencing emotional overload, body-related symbolisation of emotions, and self-control. In particular, experiencing self-control is an important goal in the TüTASS, which is achieved by practicing behaviour and focusing on the strengths of the individual participants. At the TüTASS, the children are able to try and test different methods in a safe environment, which the participants have responded to very positively.

For the participants, the acceptance of their own emotions, the experience of emotionlessness, the imaginative symbolisation of emotions, and the experience of emotion regulation (as determined by the SEE), seems unchanged. The parents reported that many of the children had difficulties with the items in the questionnaire. We found this unsurprising since the questionnaire is recommended for subjects aged 14 and older. Due to the lack of alternatives, we also used the questionnaire for a significantly younger target group which made interpretation more difficult. We are therefore glad to have used the questionnaire with the open feedback, wherein both the children and parents reported a highly positive development in terms of emotional acceptance and dealing with feelings. The objective procedure using IDS-II also showed a significant improvement to emotion regulation of 24%. We assume that, in this respect, the effectiveness stems from a combination of body awareness exercises with the concrete confrontation of emotions, both of which are fundamental features of the TüTASS.

As in our first pilot study [33], we noted an improvement in the externalising scales of the CBCL on rule-breaking and aggressive behaviour. We attribute this positive development to the appointments related to emotions and our focus on dealing with anger. Using various exercises (particularly role-playing), the participants learned a playful method of recognising and expressing emotions, and how to influence their own emotions when dealing with others. This could be also attributed to the fact that the TüTASS does not only provide information, but practises alternative behaviour. Furthermore, our body perception exercises, and in particular the methods for tension and relaxation (including body scan, PMR, and others), could be helpful for emotion regulation and thus positively influence frustration tolerance. Due to the strengthening of self-confidence, the participants also found ways to appropriately express their emotions. Conspicuous internalising behaviours also decreased according to our data collection. As with the first training, we found improvements in the CBCL in internalising behaviour for the thinking problems scale. This could be attributed to the mindfulness exercises, which help the children relax and feel less stressed. However, it should also be mentioned here that not all children and adolescents benefit equally from the mindfulness-based exercises. It is also possible that the striking behaviours used to calm oneself may have been replaced with new methods. We were pleased to note an improvement on the withdrawn/depressed subscale. One reason for this improvement could be that friendships were formed during training, and the children met before or after the sessions to spend time together. They clearly had positive interaction experiences and seemed more open to making new contacts. It is also possible that the participants showed fewer tendencies to withdraw due to reduced family conflicts. Furthermore, we saw no change in the scales for somatic complaints. This was expected as medical complaints were not treated and the body awareness exercises seemed insufficient for improving the stated symptoms. Likewise, the CBCL scores on anxious/depressed and the SDQ data on emotional symptoms remained stable, with improvements in the SDQ being shown in the first cohort. Accordingly, it seems reasonable to more deeply deal with the topic of fear in addition to the emotion of anger. For this purpose, weekly (and thus more comprehensive) support over a year would certainly be helpful. The SDQ showed a decrease in externalising problems, especially in dealing with peers. Participation in a consistent group that shares similar experiences and challenges in everyday life, is subject to reliable rules and a fixed structure, and promotes mutual respect, joint development of solutions to problems, and purposeful interactions with each other may be significant here. In addition, we found a positive trend in behavioural issues in the SDQ by exploring items targeting anger, argument, and cooperative behaviour. The hyperactivity data remained stable compared to the first cohort, while the CBCL showed a slight improvement of attention problems. Surprisingly, the body awareness exercises seemed to have little effect in this regard, although the parents reported that the children had problems engaging in the exercises regularly at home. On the other hand, we received much positive feedback regarding hyperactivity. Moreover, the prosocial behaviour did not change according to the parent’s pre and post evaluation by the CBCL. We found positive trends for the social problem’s scales in the CBCL, and the objective procedure using IDS-II showed an improvement of 15% in the socially competent behaviour scale among the participants, despite the fact that the IDS-II’s topics were deliberately not dealt with in the training. There may be a discrepancy between the acquired ideas for action and their implementation in everyday life. As such, we recommend that future groups focus even more on prosocial behaviour.

While the children’s self-reported quality of life measured by the ILK showed a slightly positive trend, both for quality of life and for the problem score, the parents’ assessment showed no improvement in these areas. A possible explanation for this difference could be that the parents cannot perceive the positive internal changes of their children accordingly, or that the external effect is perhaps more difficult due to reduced facial expressions and less shared joy [64]. The TOMTASS also descriptively shows little improvement in parents’ ratings of their children's quality of life [17]. The KINDL data showed no change in the various domains of quality of life assessed by both children and parents. The questionnaire data on quality of life contrasted with the clearly positive open feedback from parents and participants themselves, in which both reported improvements. It is also important to reiterate that the TüTASS does not aim to train children to be completely adaptable, but rather to work together to develop an improved way of dealing with special features. Based on this, and due to the constant but small-step changes over the year of the intervention, it could be difficult for the caregivers to consciously recognise and reflect on these changes in everyday life. Nevertheless, the parents’ ILK data showed a significant reduction in the parental burden of the child's ASD.

In contrast, the parents’ EBI data did not change, but showed a reduction in the perceived stress in relation to the children. The parents made active use of the accompanying parents' evenings and rated this as positive. In the open feedback questionnaire, the parents reported having also learned to take better care of themselves. The combination of theoretical input, the imparting of concrete knowledge, and action strategies on the one hand, and peer exchanges and the feeling that other families have similar challenges in everyday life on the other could be responsible for these positive effects. In terms of content, we relied on the existing parent training programmes FAUT-E [65] and FEETASS [66]. At parents’ evenings, self-care was often cited as an issue. It would certainly make sense to expand upon this side of the programme and give parents more possibilities for exchanges with others in dealing with children with autism. It could also be helpful to install a regular offer for parents, which encourages reflection through practical exercises, and focuses on body awareness exercises and mindfulness. To achieve clearer relief, it may be necessary to support the parents’ willingness to make efforts through such appropriate measures as family support.

Our study is not without limitations. Since the first version of the training was revised and extended, this is again a pilot study. Accordingly, we accept, and would like to discuss, the following limitations. We consider the small sample sizes and the lack of a control group the major limitations. Statistical data analysis was limited with the inclusion of 27 children in the study and missing questionnaires further reduced the strength of our study. Therefore, a continuation of the project, and thus an increase of sample size, is essential. Data from the upcoming participants will serve to increase the accuracy of our statements. The lack of a control group also raises the possibility that external positive influences may have altered participants’ symptom severity. Attempts were made to contain such effects by not allowing participants to participate in any other therapeutic interventions during TüTASS. But it must be emphasized that the results of this study are only preliminary and a randomized controlled trial would provide deeper and more valid insights. Although the results of the TüTASS suggest a positive change during the training, a further investigation of the TüTASS should take place in the future, taking into account a control group. Another limitation is that we did not have sufficient resources to re-diagnose all participants using ADOS and ADI-R before participation, but instead relied on the diagnosis of the pre-treatment specialist. For participants with whom ADOS diagnostics were not performed, we used SRS and MBAS to verify the assigned diagnosis. For one participant who did not meet the criteria, an expert judgment was consulted. Given sufficient time and resources, a detailed re-diagnosis would certainly be useful. We are pleased to have added objective testing to our data collection with the IDS-II, but the bulk of the data consisted of observations on self-assessments by the parents and patients themselves. The assessment in the IDS-II test was conducted by people who were not involved in the training but, due to staff randomisation of the test administration, there is no interrater reliability in collecting data using the IDS-II. Also, it would have certainly been useful to use the ADOS to assess behavioural changes at the end of the training, which we were not able to do due to time constraints. This should be considered in future studies. Furthermore, it should be mentioned that six patients were not subjected to IQ testing before the training. Thus, it is not guaranteed that they actually have the required IQ of over 70. However, based on the perception of the therapists and the school types attended by the participants, it can be assumed that the IQ was within the normal range. Another limitation of our study is that the comorbidities of the participants were not clearly collected. Although the parents were asked about this before the start of the training, the information was incomplete and therefore not usable for the study. In the future, comorbidities must be systematically recorded to better understand individual disease courses and their changes. Another limitation relates to the uneven gender distribution, possibly reflecting the higher proportion of males with autism [1, 5]. Moreover, in developing the intervention, we are bound to literature that is specifically based on the expression of autistic symptomatology in males. Therefore, we can only make insufficient statements as to whether our intervention also adequately covers the needs of autistic females. Further, it should be mentioned that in previous research mindfulness training was mostly applied in individual settings and there are hardly any findings on its application in group therapies. However, from our clinical expertise, we have found that mindfulness exercises in a group setting have the advantage of sharing and practicing them more in social settings. It is clear that not all participants benefited equally. However, it was clear from interviews with participants and their parents that the mindfulness exercises were found to be helpful. Further research is needed in the future to investigate the effectiveness of mindfulness exercises in group settings and in specific subgroups of autistic children and adolescents.

It should also be noted that the evaluation was performed on the basis of the primary and secondary outcomes without alpha error correction. Since this was the first evaluation of our treatment and to detect all effects, an exploratory examination of the secondary outcomes was performed. Therefore, no alpha error correction was performed at this point. However, for future evaluation of the training, a larger sample with alpha error correction should be used. Considering an alpha level reduction on *p* = 0.001 to reduce alpha inflation for all secondary outcomes, only the significant decrease of CBCL rule-breaking and aggressive behaviour would remain, pointing to a good influence of the training on important aspects of social behaviour.

Nevertheless, we achieved our overall goal, which was to demonstrate therapy- and group-independent efficacy and patient acceptance of this group programme. The TüTASS represents a theoretically substantiated and currently discussed therapeutic approach that addresses the real deficits of children with ASD. With the application of the revised and extended TüTASS in five groups, the feasibility of the training could be proven again. Due to the great interest of the participants in further group meetings, we decided to permanently offer the TüTASS Extended for all participants in additional groups. Moreover, the interest in participating now extends far beyond the borders of Tübingen. To investigate how children benefit from the training in the long term, we are currently evaluating the revised training with further survey assessments.

## Data Availability

Data is available from the authors upon request.
